# The Mechanism of Heterogeneous Beta-Lactam Resistance in MRSA: Key Role of the Stringent Stress Response

**DOI:** 10.1371/journal.pone.0082814

**Published:** 2013-12-09

**Authors:** Choonkeun Kim, Michael Mwangi, Marilyn Chung, Catarina Milheirço, Herminia de Lencastre, Alexander Tomasz

**Affiliations:** 1 Laboratory of Microbiology, The Rockefeller University, New York, New York, United States of America; 2 Department of Veterinary and Biomedical Sciences, Penn State University, University Park, Pennsylvania, United States of America; 3 Laboratory of Molecular Genetics, Instituto de Tecnologia Química e Biológica da Universidade Nova de Lisboa, Oeiras, Portugal; National Institutes of Health, United States of America

## Abstract

All methicillin resistant *S. aureus* (MRSA) strains carry an acquired genetic determinant – *mecA* or *mecC* - which encode for a low affinity penicillin binding protein –PBP2A or PBP2A′ – that can continue the catalysis of peptidoglycan transpeptidation in the presence of high concentrations of beta-lactam antibiotics which would inhibit the native PBPs normally involved with the synthesis of staphylococcal cell wall peptidoglycan. In contrast to this common genetic and biochemical mechanism carried by all MRSA strains, the level of beta-lactam antibiotic resistance shows a very wide strain to strain variation, the mechanism of which has remained poorly understood. The overwhelming majority of MRSA strains produce a unique – heterogeneous – phenotype in which the great majority of the bacteria exhibit very poor resistance often close to the MIC value of susceptible *S. aureus* strains. However, cultures of such heterogeneously resistant MRSA strains also contain subpopulations of bacteria with extremely high beta-lactam MIC values and the resistance level and frequency of the highly resistant cells in such strain is a characteristic of the particular MRSA clone. In the study described in this communication, we used a variety of experimental models to understand the mechanism of heterogeneous beta-lactam resistance. Methicillin-susceptible *S. aureus* (MSSA) that received the *mecA* determinant in the laboratory either on a plasmid or in the form of a chromosomal SCC*mec* cassette, generated heterogeneously resistant cultures and the highly resistant subpopulations that emerged in these models had increased levels of PBP2A and were composed of bacteria in which the stringent stress response was induced. Each of the major heterogeneously resistant clones of MRSA clinical isolates could be converted to express high level and homogeneous resistance if the growth medium contained an inducer of the stringent stress response.

## Introduction

In most clinical isolates of methicillin-resistant *Staphylococcus aureus* (MRSA) resistance to the beta-lactam family of antibiotics is expressed in a peculiar – heterogeneous – fashion. Such hetero-resistant MRSA, grown from single-cell inocula produce cultures in which the majority of cells exhibit only low or moderate level of antibiotic resistance – sometimes barely above the MIC threshold of a susceptible strain. However, subpopulations of highly resistant cells are also present in these cultures with MIC values and frequencies that are characteristic of the particular MRSA strain [1]. Quantitative analysis of such MRSA cultures through the method of population analysis can provide phenotypic profiles that are stable characteristics of the particular MRSA strain [1,2]. Historically early MRSA isolates from the UK and from Denmark all showed heteroresistant population analysis profiles (PAPs) [3] and heteroresistant MRSA clones continue to be frequently identified among contemporary isolates of MRSA both from hospitals and also from the community [4]. While MRSA clones expressing methicillin resistance in a more homogeneous fashion have also been identified [4] – such as the “Iberian” clone (ST247-SCC*mec* IA) [5–7], the “Brazilian” clone (ST239-SCC*mec* III) [8,9], and the “EMRSA-16” clone (ST36-SCC*mec* II) [10,11] – a correlation between the resistance phenotype and any particular epidemic potential of these clones has remained unclear.

In a recent effort to better understand the mechanism of expression of beta-lactam resistance, we described a laboratory model system in which plasmid-borne copies of the *mecA* determinant were introduced into the background of the fully sequenced MSSA strain 476 [12]. Introduction of the plasmid-borne *mecA* into strain 476 generated a heteroresistant derivative named 476(*pmecA*) in which the majority of bacteria expressed only very low oxacillin MIC value (0.75 μg/ml) but an apparently single subpopulation of highly resistant mutant bacteria - named 476^mut^(*pmecA*) - was also present with an oxacillin MIC of 800 μg/ml and a frequency of about 10^-4^. More detailed analysis of the poorly resistant majority cells (476(*pmecA*)) and the highly resistant mutant subpopulation (476^mut^(*pmecA*)) indicated that the genetic factor responsible for the vastly different resistance level resided in the genetic background of the cells and comparison of full genome sequences of the majority and the highly resistant minority bacteria identified a single genetic element – the S. *aureus relA* gene – as the critical determinant defining the oxacillin MIC values [13]. Specifically, bacteria that were able to express high-level resistance from the plasmid-borne *pmecA* carried a single mutation in the *relA* gene that introduced a stop codon into the gene after the SYN domain, thus producing a truncated *relA* from which the controlling TGS and ACT domains were missing [13]. Such a mutant would be expected to produce (p)ppGpp - the gene product of *relA* [14,15] - in a constitutive fashion triggering the stringent stress response in the mutant bacteria. Introduction of plasmid-born copies of *mecA* into the *relA* mutant 476^mut^(*pmecA*) carrying the *relA* mutation produced bacteria with an approximately 4-fold excess in the cellular amounts of (p)ppGpp – as compared to the isogenic strain 476(*pmecA*) which carries the wildtype *relA* gene [13]. Cultures of 476^mut^(*pmecA*) also exhibited high and homogeneous resistance which was shared by all cells in the culture [13]. 

The purpose of the study described in this communication was to follow up these findings in several directions.

i) Using the original model system with the plasmid-borne *mecA* gene we determined biochemical correlates of antibiotic resistance as well as transcription of the *mecA* gene and its translation to PBP2A.
ii) We extended the observations to another laboratory model strain RUSA11 in which the entire SCC*mec* I complex (instead of a plasmid-borne *mecA*) from MRSA strain COL was introduced into the background of the MSSA strain RN2677 resulting in an MRSA construct – named RUSA11 – that expressed heterogeneous oxacillin resistance but could be induced to produce homogeneous and highly resistant phenotype by mupirocin – an agent capable of inducing the stringent response in the bacteria [16–18]. Inclusion of mupirocin in the antibiotic containing plates used for the determination of PAPs of strain RUSA11 or the clinical heteroresistant strain MW2 - produced cultures that were highly and homogeneous resistant to oxacillin and showed increased production of the resistance protein PBP2A.
iii) We were also able to demonstrate that representatives of major heteroresistant MRSA clones frequently recovered from the clinical environment were able to express high and homogeneous resistance to beta-lactam antibiotics if the stringent stress response was induced in the bacteria.

## Materials and Methods

### Bacterial strains and growth conditions

Strains 476, 476(*pmecA*), 476^mut^, and 476^mut^(*pmecA*) [13] were incubated in tryptic soy broth (TSB, Difco Laboratories) containing 20 µg/ml of chloramphenicol at 30 °C to maintain the temperature-sensitive plasmid pSTSW2C. All other *S. aureus* cultures were routinely grown at 37 °C in TSB or on tryptic soy agar (TSA, Difco Laboratories). The bacterial strains and a plasmid used in this study are described in Table 1. Oxacillin (various concentrations) or/and mupirocin (0.03 µg/ml) was added in the media to determine resistance phenotypes.

**Table 1:** Strains and plasmids.

Strains and plasmid	Description	Reference
***S. aureus* strains**		
476	Methicillin-susceptible *S. aureus* (MSSA) isolated in 1998 from a 9-year-old boy with community-acquired primary upper tibial oxteomyelitis and bacteremia	[12]
476(*pmecA*)	Strain 476 carrying the plasmid-borne *mecA* region (pSTSW2C); (Resistance phenotype heterogeneous)	[13]
476^mut^(*pmecA*)	476pmecA having mutations in the *relA* gene; (Resistance phenotype homogeneous)	[13]
476^mut^	476^mut^(*pmecA*) from which the *pmecA* was removed by growth at a temperature non permissive for plasmid replication	[13]
RN2677	Non-lysogenic derivative of NCTC8325; β-lactamase negative; methicillin-susceptible; novobiocin- and rifampin-resistant	[25,26]
RUSA11	RN2677 carrying *S. aureus* COL SCC*mec* in the chromosome; (Resistance phenotype heterogeneous)	This study
RUSA11^HR^	RUSA11 picked at 100 µg/ml of oxacillin; (Resistance phenotype homogeneous)	This study
MW2	Highly virulent community-acquired MRSA isolated in 1998 in North Dakota, USA (Resistance phenotype heterogeneous)	[27]
MW2^HR^	Colony of MW2 picked from an agar plate containing 100 µg/ml of oxacillin; (Resistance phenotype homogeneous)	This study
**Plasmid**		
pSTSW2C	pSPT181C with 3,737-pb PCR product of *S. aureus* COL *mecA* region; chloramphenicol resistant	[21]

### Antibiotic susceptibility tests

The susceptibilities of *S. aureus* strains to oxacillin were examined by population analysis profiles (PAPs) [1,2,19] and/or by Etest (bioMériux, Inc.). The Etest was performed by spreading a small aliquot of overnight cultures diluted to an OD_620_ of 0.08 on TSA plates containing 0.03 µg/ml of mupirocin or 1 mg/ml of serine hydroxamate (SHX) if necessary, followed by placing oxacillin Etest strips on the plates. Minimal inhibitory concentration (MIC) values of oxacillin were evaluated after 48 h incubation at 30 °C and/or 37 °C. Colony forming units (CFU) were counted after 48 h of incubation of the plates at 30 °C or 37 °C.

### Preparation of staphylococcal membrane proteins

Membrane fractions were prepared from the S. *aureus* strains following the method described previously [20] with slight modification. Briefly, in order to determine the amounts of PBP2A associated with the bacterial plasma membrane, *S. aureus* strains 476, 476(*pmecA*), 476^mut^, and 476^mut^(*pmecA*) were grown at 30 °C in 200 ml of TSB. All other *S. aureus* strains were grown at 37 °C. Mupirocin (0.03 µg/ml) and/or oxacillin (0.5 µg/ml) were added to the growth media to test the effect of these agents on the amounts of PBP2A produced by the bacteria. All strains were harvested at OD_620_ of 0.5, washed and resuspended in 3 ml of 20 mM Tris-HCl, pH 7.6 containing 1× Halt protease inhibitor cocktail (Thermo Fisher Scientific, Inc.), 10 mM MgCl_2_ 100 µg/ml lysostaphin, 50 µg/ml lysozyme, 50 µg/ml DNase I, and 50 µg/ml RNase A. Cells were incubated at 37 °C for 30 min and disrupted by sonication with pulse of 40% output for 5 min. The suspensions were centrifuged at 7,000 × g for 20 min to remove unbroken debris, and the supernatants were transferred to fresh ultracentrifuge tubes. Membrane fractions were collected by centrifugation at 100,000 × g for 1 h. The collected membranes were resuspended in 20 mM Tris-HCl, pH 7.6 and stored at -70 °C. The concentration of total membrane proteins was determined by the BCA assay.

### Western blotting

Western blotting was performed for detection of PBP2A in membrane preparations. The procedure was as described previously [20–22] with a few modifications. For strains 476, 476(*pmecA*), 476^mut^, and 476^mut^(*pmecA*) 50 µg of the membrane proteins were loaded to SDS-PAGE while 100 µg of the protein was used for all MW2 strains. The primary antibody (rabbit anti-PBP2A antibody) was used with dilution of 1:15,000, and the secondary antibody was the HRP-coupled anti-rabbit antibody (0.5 mg/ml; PerkinElmer.), which was diluted 1:5,000. The ChromPure human IgG Fc fragment (Millipore) was added at a final concentration of 3 µg/ml in order to prevent the secondary antibody from non-specific binding. Pierce ECL 2 (Thermo Fisher Scientific, Inc.) was used for the substrate of HRP in order to visualize PBP2A bands both with chemiluminescence and with fluorescence, which were scanned on a Typhoon9400 Image Scanner. 

### Northern blotting

Northern blotting was carried out to determine the effect of oxacillin and mupirocin on *mecA* gene transcription. Strains 476, 476(*pmecA*), 476^mut^, and 476^mut^(*pmecA*) were grown overnight and then diluted 1:100 in fresh TSB with/without mupirocin and/or oxacillin. The bacterial cultures were collected for extraction of RNA when OD620 was 0.7. RNA was isolated using RNeasy kit (QIAGEN) following the manufacturer’s instruction. RNA (2.5 µg) was electrophoresed on a 1.2% agarose-0.7 M formaldehyde gel in MOPS (morpholinepropanesulfonic acid) running buffer. RNA was transferred to a nylon membrane using TURBOBLOTTER (Whatman) and fixed by UV cross-linking, and then the ethidium bromide-stained RNA was visualized by UV illumination of the transfer membrane. The probe used for detecting the mecA transcripts was a 500-bp PCR product of chromosomal DNA from *S. aureus* COL. The probe was labeled using Amersham Ready-To-Go DNA Labelling Beads (GE Healthcare) with [α-^32^P]dCTP. The probe was hybridized with RNA at 65 °C in a SDS hybridization solution containing 5X SSPE (Invitrogen), 5X Denhardt’s reagent (Invitrogen), 0.5% SDS and 100 µg/mL Salmon sperm DNA (Invitrogen). The washed membrane was exposed to a phosphor screen for 5 hours and scanned on a Typhoon9400 Image Scanner.

## Results and Discussion

**Figure 1:**
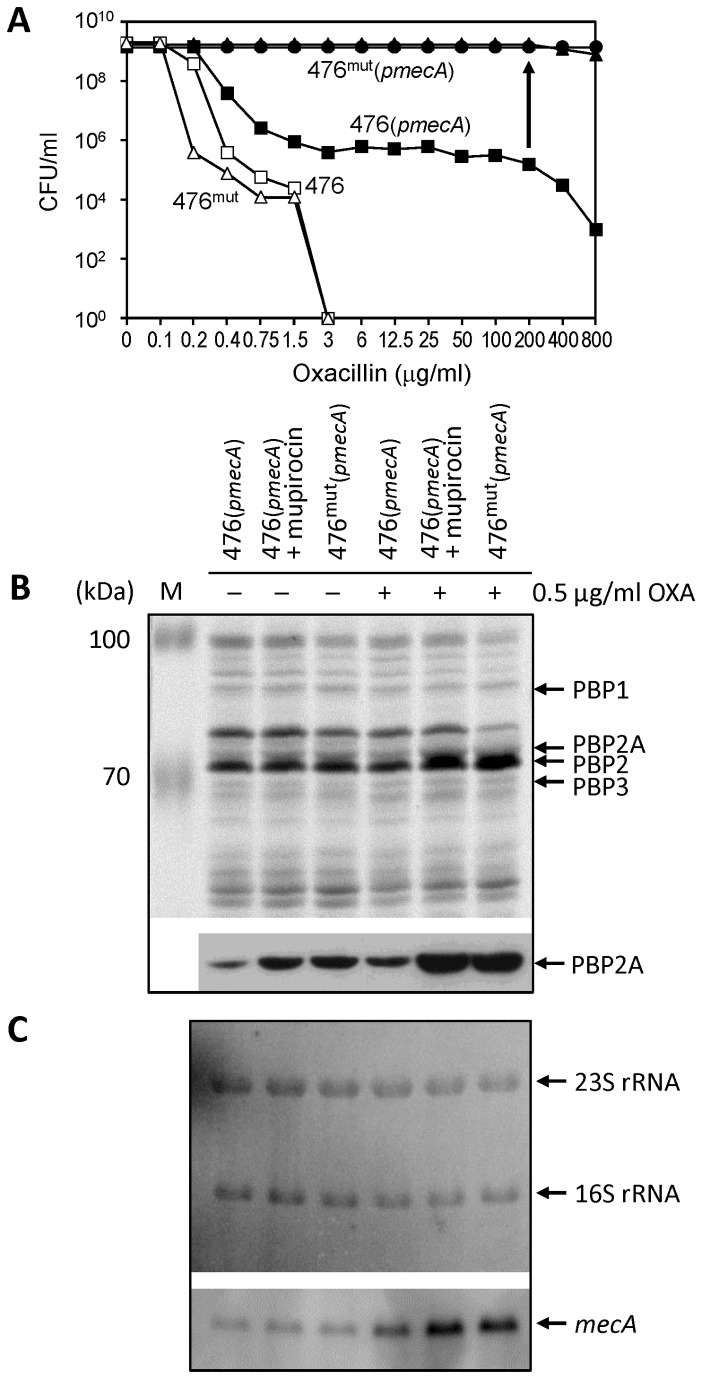
Correlation between oxacillin resistance and the level of transcription and translation of *mecA* – in the 476(pmecA) model system. (**A**) Susceptibility of strains and constructs to oxacillin was determined by population analysis. Population Analysis Profiles (PAPs) are shown for the following: MSSA strain 476 (□); 476(pmecA) (■) (strain 476 carrying plasmid borne copies of *mecA*); 476^mut^(pmecA) (▲) (culture of a highly resistant colony picked from agar containing 200 µg/ml oxacillin); 476^mut^ (△) (derivative of 476^mut^(pmecA) “cured” of the plasmid–borne *mecA*); 476(pmecA)“induced” (●) (476(pmecA) plated on oxacillin containing agar supplemented with 0.03 µg/ml mupirocin). (**B**) SDS-PAGE analysis of membranes isolated from strain 476 and its derivatives. The relative amounts of PBP2A were determined by Western Blotting using a monoclonal antibody prepared against PBP2A. Expression of PBP2A was induced in 476(pmecA) by oxacillin (lanes 4-6). “M” indicates molecular size marker. (**C**) Northern blotting was used to assay transcription of *mecA*. Transcriptions of 16S and 23S rRNA were used as controls.

Figure 1A reproduces the antibiotic resistance phenotypes described recently in a model system in which the fully sequenced and antibiotic-susceptible *S. aureus* strain 476 was transduced to carry copies of the plasmid-borne *mecA* [13]. Transductants of the strain – named 476(*pmecA*) – showed a heterogeneous phenotype: the majority of cells expressed only low-level oxacillin resistance (MIC of about 0.75 μg/ml) but highly resistant bacteria were also present with an approximate frequency of 10^-4^. Using as inoculum for an over-night culture a colony picked from the agar plate containing 200 μg/ml of oxacillin, we were able to produce a highly and homogeneously resistant mutant culture – named 476^mut^(*pmecA*). 

Comparison of the genetic backgrounds of the two strains – 476 and 476^mut^ – by full-genome sequencing – identified a single mutation in the *relA* gene that was responsible for the highly resistant phenotype [13].

The population analysis profile of the majority cells 476(*pmecA*) also became homogeneously and highly resistant if the oxacillin containing plates were supplemented with sub MIC concentrations of mupirocin – an isoleucine homolog that inhibits isoleucyl-tRNA synthetase (Figure 1A). 

Several lines of evidence indicate that this effect of mupirocin was related to the induction of the stringent stress response in the bacterium: the growth rate of strain 476(*pmecA*) decreased gradually with increasing concentrations of mupirocin in the medium – from a doubling time of 22 minutes in the absence of mupirocin to 32, 46 and 64 minutes – respectively – when the growth medium was supplemented with mupirocin at concentrations of 0.03, 0.045 and 0.06 μg/ml of the agent. Addition of mupirocin to the growth medium was also shown to cause accumulation of ppGpp and ppGppp in the cells [13]. 

As a further confirmation for the involvement of the stringent stress, the oxacillin MIC values of strain 476(*pmecA*) and the heteroresistant clinical isolate MW2 were also determined in a medium supplemented with serine hydroxamate, a serine analog that inhibits seryl-tRNA synthetase [23,24] and provokes the stringent response due to serine starvation. In the presence of serine hydroxamate (1 mg/ml) the MIC value of 476(*pmecA*) increased from 0.75 up to 125 μg/ml and the oxacillin MIC of strain MW2 increased from 0.5 up to 250 μg/ml. 

In the experiments to be described next we followed up these findings by biochemical analysis of the 476(*pmecA*) constructs. Figure 1B shows SDS-PAGE analysis and Western blotting to determine the protein profiles and the relative amounts of PBP2A produced by the bacteria. Figure 1C shows the level of transcription of *mecA* in each of the bacterial constructs. 

The SDS-PAGE patterns of cultures were very similar (Figure 1B). On the other hand, the relative amounts of the PBP2A – determined by Western blotting - showed parallels with the relative antibiotic resistance levels of the bacteria. Thus, 476(*pmecA*) grown in the presence of mupirocin and 476^mut^(*pmecA*) both showed substantially increased amounts of PBP2A as compared to strain 476(*pmecA*). 

The relative amounts of PBP2A among the same strains showed even larger differences when the titration of PBP2A was repeated in the presence of sub-MIC concentrations of oxacillin (0.5 μg/ml), which was used to release the control of transcription of *mecA* by the *bla* system that is present in strain 476 on a penicillinase plasmid [12]. This was confirmed by the large increase in the transcription of *mecA* in each one of the cultures tested in the presence of 0.5 μg/ml of oxacillin (Figure 1C). 

Addition of mupirocin to 476(*pmecA*) caused increase in the amount of PBP2A, which was similar to the relative amounts of PBP2A found in strain 476^mut^(*pmecA*) (Figure 1B). 

In the experiments described in Figure 1A-C the model system used was the beta-lactam susceptible strain *S. aureus* 476 into which plasmid-borne copies of *mecA* gene were introduced [13]. In order to test the validity of the observations obtained in this model system, we next extended the tests to a construct in which the source of the resistance gene was not the plasmid-borne *mecA* determinant but an entire SCC*mec* type I cassette of the MRSA strain COL, which was transferred – by genetic transformation – into the beta-lactam susceptible *S. aureus* strain RN2677, a derivative of the methicillin-susceptible isolate 8325, a strain that do not carry a penicillinase plasmid [25,26]. 

**Figure 2:**
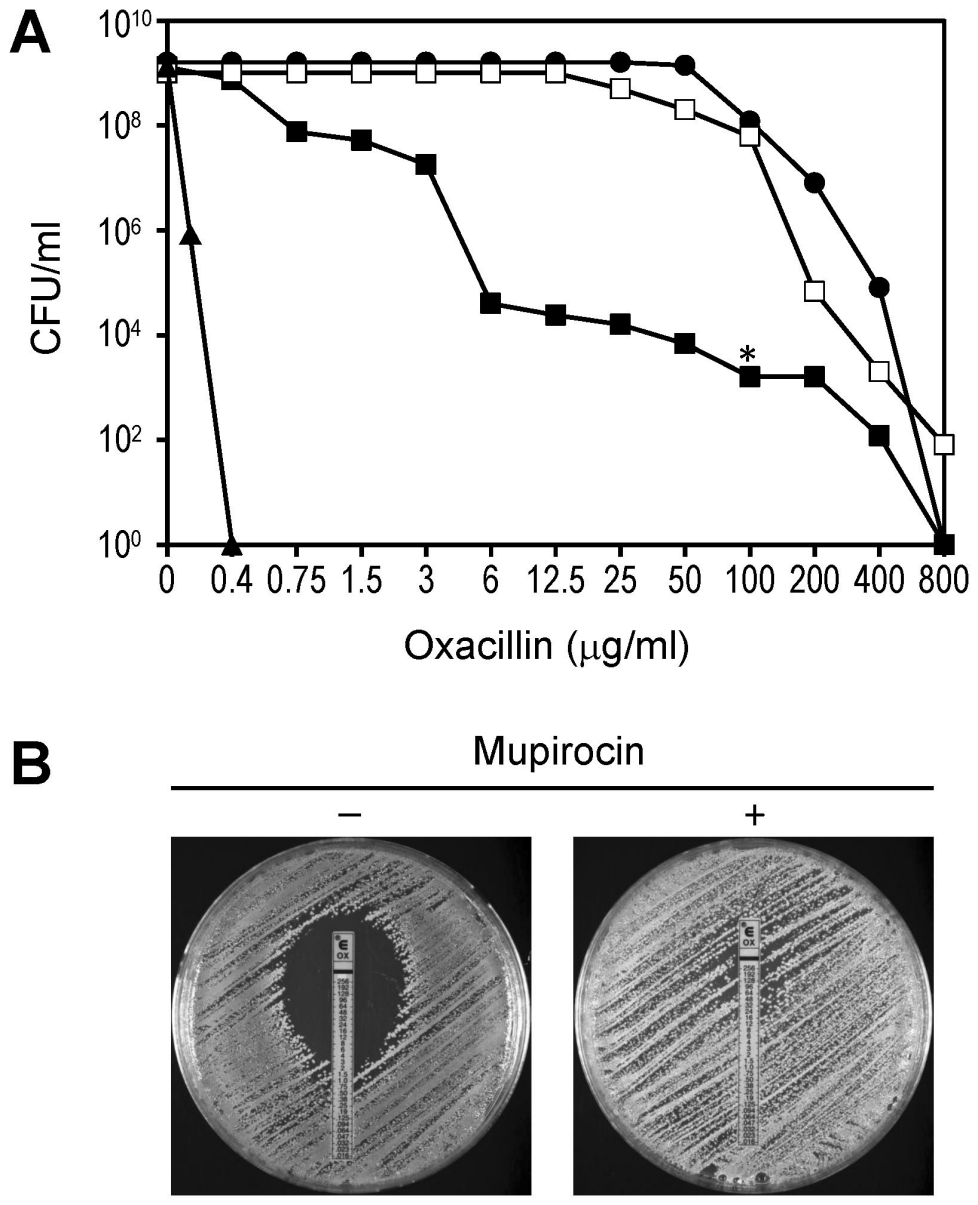
Heterogeneous expression of oxacillin resistance in the laboratory strain RUSA11. (A) Oxacillin resistance level of the strains was determined by population analysis profiles (PAPs). PAP of the susceptible *S. aureus* recipient strain RN2677 (▲), oxacillin resistant strain RUSA11 (■); RUSA11 tested on oxacillin containing plates supplemented with sub-MIC concentration of mupirocin (□); culture of a highly resistant colony of RUSA11 (*) picked from a plate containing 100 µg/ml oxacillin (●). (B) Oxacillin susceptibility of RUSA11 determined by the E-test on agar plates with or without supplementation of the agar by mupirocin (0.03 µg/ml).

Figure 2A shows the population analysis profiles (PAPs) of the susceptible-recipient strain RN2677; the oxacillin-resistant transformant strain RUSA11; and the progeny of a highly resistant colony of RUSA11 that was picked from an agar plate containing 100 μg/ml of oxacillin (see * in Figure 2A). Bacteria with high-level resistance were present in the original RUSA11 culture with an approximately frequency of 10^-5^. Figure 2A also shows the PAP of RUSA11 determined on agar plates in which the oxacillin concentrations were supplemented with a single sub-MIC concentration (0.03 μg/ml) of mupirocin. 

Figure 2B shows the results of E-tests performed on the RUSA11 construct in the presence and absence of mupirocin in the agar plate. 

The results of experiments summarized in Figure 2 clearly validate the conclusions obtained earlier using the plasmid-borne *mecA* model – namely, the sensitive dependence of the beta-lactam MIC value on the genetic background of the S. *aureus* strain – more specifically by the status of the *relA* determinant [13]. 

The purpose of the next series of experiments was to extend the basic observations made with laboratory models to clinical isolates of heteroresistant MRSA. As a first strain we used the fully sequenced clinical MRSA strain MW2 [27], which is a close relative of the MSSA strain 476, used in the model experiments. 

**Figure 3:**
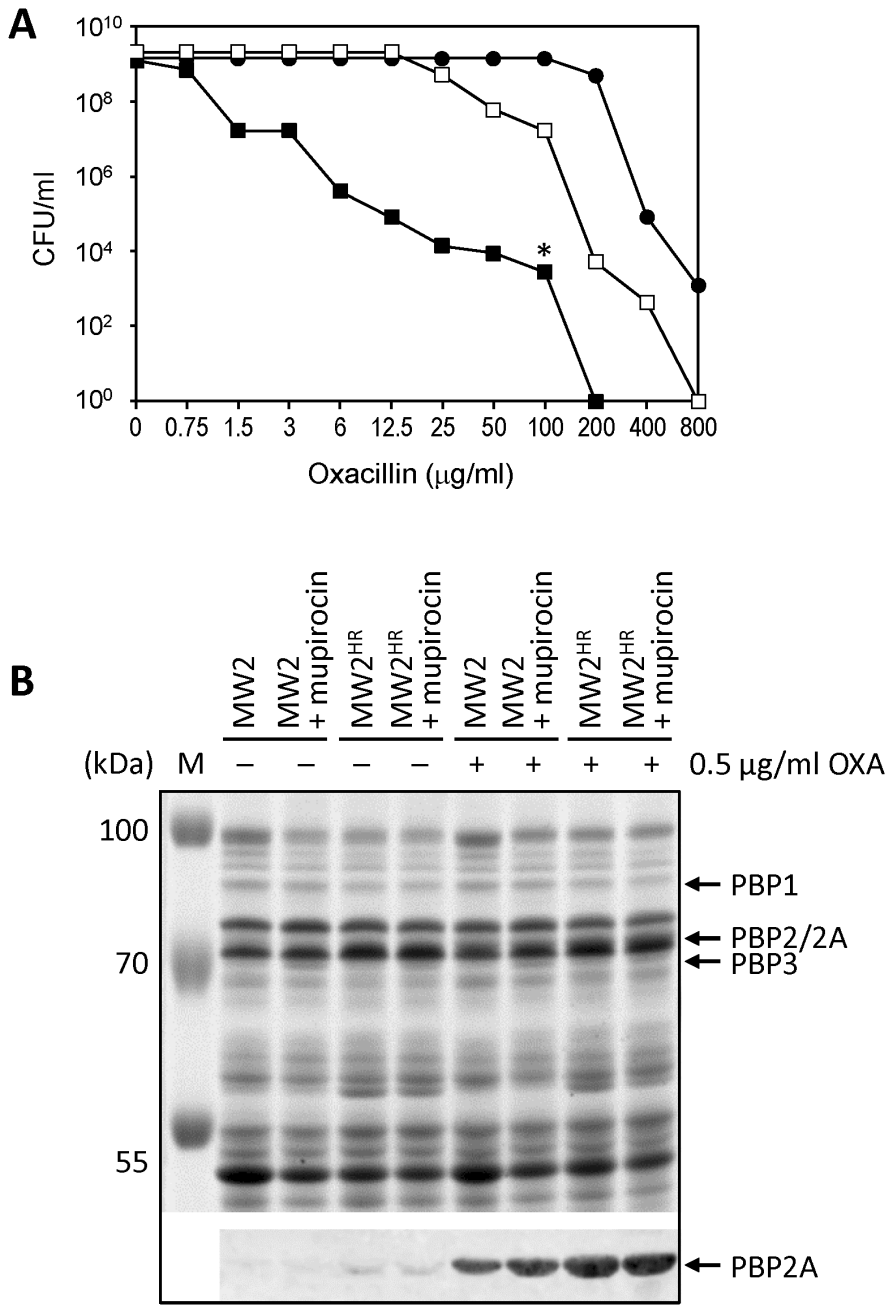
Conversion of heterogeneous oxacillin resistance to high and homogenous resistance – in the clinical MRSA isolate MW2. (**A**) Population analysis profiles are shown for the following strains. MW2 tested on agar plates containing oxacillin (■) or on agar plates also supplemented with sub-MIC concentrations of mupirocin (□). MW2 ^HR^ (highly resistant derivative of MW2) (●) - MW2^HR^ was generated from a colony of MW2 picked from agar containing 100 µg/ml of oxacillin (*). (**B**) SDS-PAGE analysis of membranes prepared from MW2 and its derivatives. Analyses were performed as described in Methods and in the legend to Figure 1B. Relative amounts of PBP2A were determined by Western blotting with a monoclonal antibody against the protein. PBP2A was only detectable in strains that were induced by sub-MIC concentrations (0.5 µg/ml) of oxacillin which was needed to release inhibition of *mecA* transcription by the *blaI-R* regulatory genes carried on a penicillinase plasmid which is present in strain MW2.

Figure 3A shows the population profiles (PAP) of strain MW2 determined with and without the presence of sub-MIC concentration of mupirocin in the oxacillin-containing plates. Also shown is the PAP of a culture generated from a colony of MW2 that was able to grow in the presence of 100 μg/ml of oxacillin. 

Figure 3B shows the SDS-PAGE profiles of the strains and determination of the relative amounts of PBP2A by Western blotting in the presence or absence of sub-MIC concentrations of oxacillin, which was used to release the transcriptional control of *mecA*. While strain MW2 carries an SCC*mec* type IV that lacks an active *mecI*/*mecR* system, the same strain carries a penicillinase plasmid equipped with the *blaI*/*blaR* genes, which are known to also control the expression of the chromosomal *mecA* [27–29]. 

Figure 3B shows that the amounts of PBP2A were greatly increased in cultures induced by sub-MIC concentrations of oxacillin and within that group of cultures the amounts of PBP2A were further increased in the highly-resistant subpopulations MW2^HR^ as well as in both cultures MW2 and MW2^HR^ that were treated with mupirocin. 

**Figure 4:**
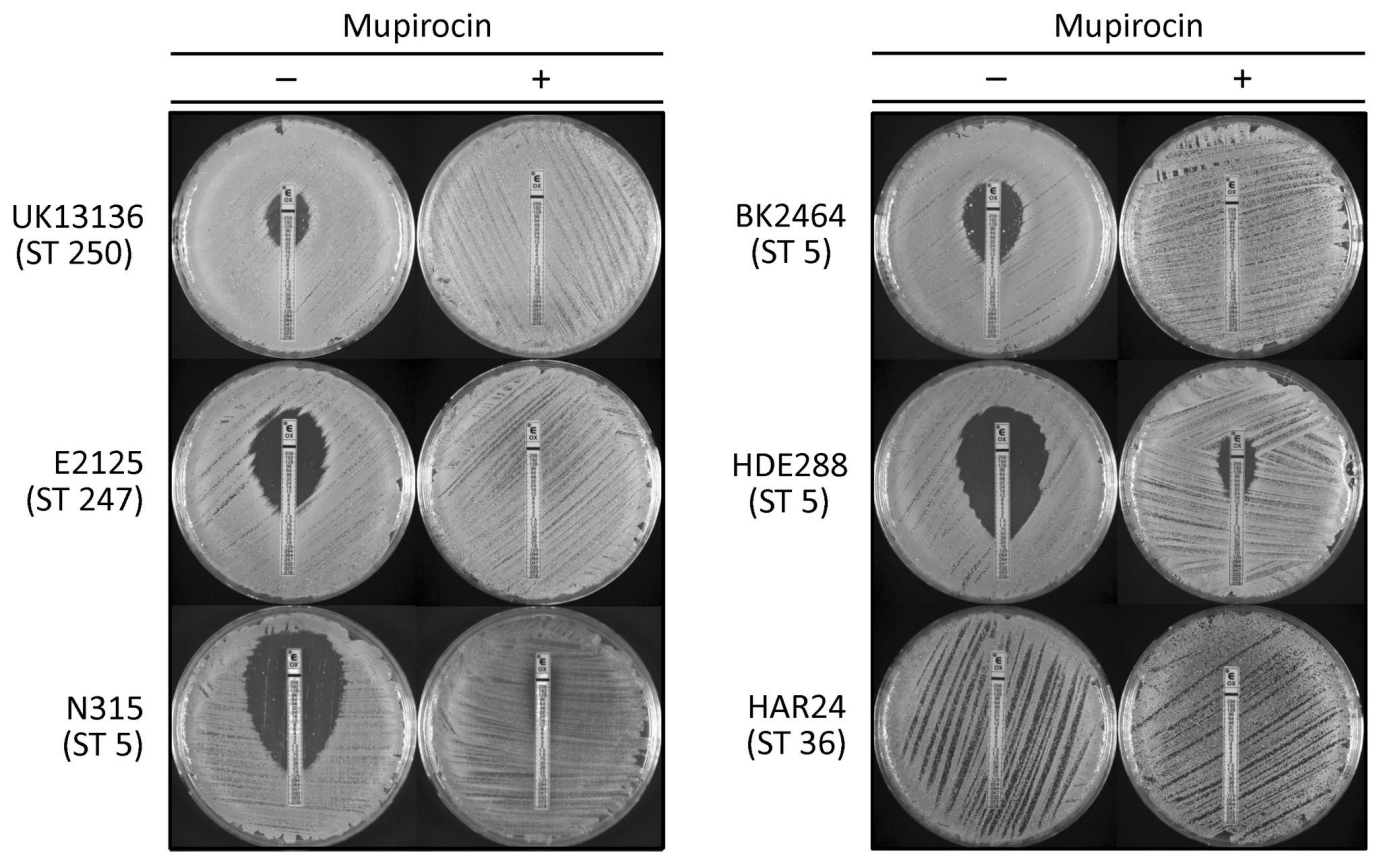
Conversion of the heterogeneous oxacillin resistance phenotypes to homogeneous and high level resistance – in clinical MRSA isolates representing major MRSA clones. Etests for oxacillin resistance were done with or without induction by a sub-MIC concentration (0.03 µg/ml) of mupirocin added to the agar medium.

In order to further extend the validity of conclusions obtained with the laboratory strains, we next tested the oxacillin MIC values of six major MRSA clones of different sequence types recovered from clinical samples. Figure 4 and Table 2 show that similar to the observations made with the laboratory strains, in the clinical isolates too, the low-level and heterogeneous resistance could be converted to high and homogeneous resistance to oxacillin in the presence of sub-MIC concentrations of mupirocin, i.e., by simultaneous triggering of the stringent response in the bacteria.

**Table 2:** Effect of mupirocin on the beta-lactam antibiotic resistant phenotype of clinical MRSA isolates.

Isolation				Molecular typing**^*a*^**				MIC of oxacillin (µg/ml)**^*b*^**			
Strain	Origin	Year		Clonal type	MLST ST	SCC*mec*		– mupirocin		+ mupirocin	
UK13136	UK	1961		Archaic	250	I		24	(heterogeneous)**^*c*^**	>256	(homogeneous)
E2125	Denmark	1964		Archaic	247	I		4	(heterogeneous)	>256	(homogeneous)
N315	Japan	1982		NY/JP	5	II		2	(heterogeneous)	>256	(homogeneous)
BK2464	United States	1996		NY/JP	5	II		8	(heterogeneous)	>256	(homogeneous)
HDE288	Portugal	1996		Pediatric	5	VI		0.25	(heterogeneous)	24	(homogeneous)
MW2	United States	1998		CA-MRSA	1	IVa		1	(heterogeneous)	>256	(homogeneous)

^a^ Abbreviations: NY/JP, New York/Japan; CA-MRSA, community-acquired MRSA; MLST, multilocus sequence typing; .

ST, sequence type; SCC*mec*, staphylococcal chromosomal cassette *mec*; MIC, minimum inhibitory concentration.

^b^ MIC values were examined by Etest.

^c^ Resistance phenotypes were determined by PAP as described in the parentheses.

**Figure 5:**
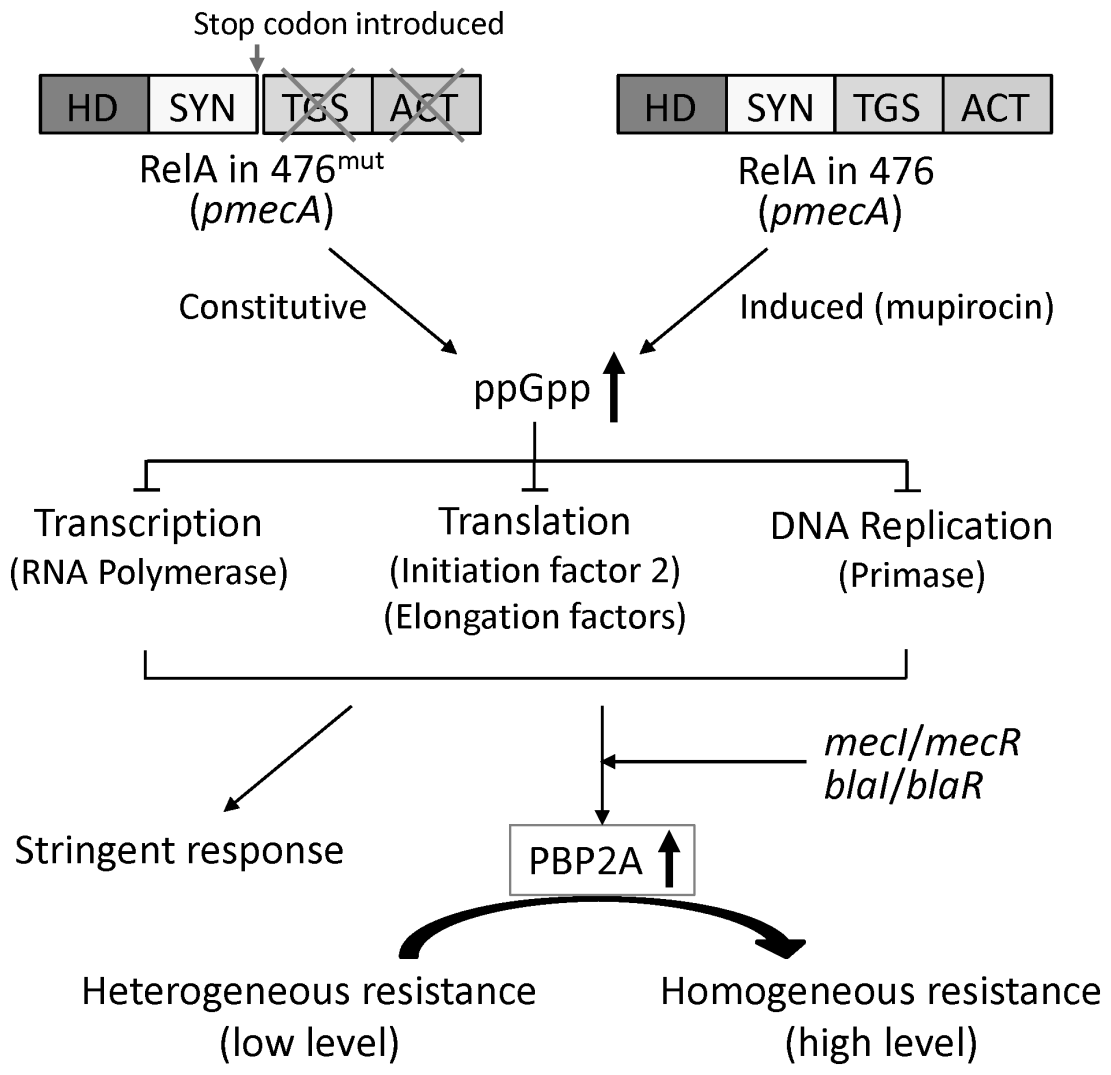
Model for the role of the *relA* gene in the phenotypic expression of oxacillin resistance.

Figure 5 summarizes the proposed model for the critical contribution of the *relA* system to the antibiotic resistance phenotype of MRSA strains. According to our model, expression of high-level resistance requires the triggering of the *relA* system to invoke a stringent stress response in highly resistant MRSA. In the original model system using plasmid-borne *mecA*, we were able to identify in highly resistant bacteria a mutation in the *relA* gene, which causes uncontrolled constitutive functioning of the *relA* system including the continued production of (p)ppGpp, which induces the stringent response in the bacteria [13]. The activation of the stringent response is known to slow-down growth and suppress production of most classes of proteins in all bacterial species examined. Interestingly, while the highly resistant subpopulations in MRSA clones are known to grow slower and bacteria in which high-level resistance is induced by mupirocin also slow-down their growth, the transcription of the foreign-borne *mecA* determinant (see Figure 1C) and the production of the resistance protein PBP2A (see Figures 1B and Figure 3) were both greatly increased under the same conditions, leading to the expression of high and homogeneous antibiotic resistance. 

Many details of this unexpected contribution of the *relA* system to antibiotic resistance remain to be further explored. Our experiments with the mupirocin-treated heteroresistant MRSA clinical strains leave little doubt that the basic model identified in the laboratory experiments are also true for heteroresistant clones of MRSA clinical isolates.

The model proposed does not imply that the highly resistant subpopulations of heteroresistant MRSA isolates each carry a mutated *relA*. Because of the central role the *relA* system plays in controlling bacterial metabolism, a large variety of mutations in genes involved in nutrient utilization and/or uptake or mutations in RNA polymerase would all be expected to trigger the stringent response. 

The nature of these specific mutations carried by the highly resistant subpopulations of MRSA clones remains to be determined. Our experiments with the model systems suggest that a common consequence of these variable mutations would be triggering the stringent response through the over-production of (p)ppGpp. 

The increase in antibiotic resistance accompanied by over-production of the PBP2A protein in a bacterium in which growth and metabolism are slowed-down and production of most “host” proteins is inhibited - is quite surprising. Our observations suggest that the transcription and translation of the foreign-borne genetic determinant *mecA* may be exempt from the control of one of the major regulatory systems of *S. aureus*, since the slow-down in growth in the highly resistant fraction of bacteria is actually accompanied by an increase in the transcription and translation of the imported resistant determinant *mecA*.
