# Correction: The Mechanism of Heterogeneous Beta-Lactam Resistance in MRSA: Key Role of the Stringent Stress Response

**DOI:** 10.1371/annotation/7775dee3-7a91-4c44-9125-c7634bf909e4

**Published:** 2014-01-02

**Authors:** Choonkeun Kim, Michael Mwangi, Marilyn Chung, Catarina Milheiriço, Herminia de Lencastre, Alexander Tomasz

The name of the fourth author is incorrect. The correct name is: Catarina Milheiriço. The correct Citation is: Kim C, Mwangi M, Chung M, Milheiriço C, de Lencastre H, et al. (2013) The Mechanism of Heterogeneous Beta-Lactam Resistance in MRSA: Key Role of the Stringent Stress Response. PLoS ONE 8(12): e82814. doi:10.1371/journal.pone.0082814. 

